# Oncosuppressor-Mutated Cells as a Liquid Biopsy Test for Cancer-Screening

**DOI:** 10.1038/s41598-019-38736-y

**Published:** 2019-02-20

**Authors:** Mohamed Abdouh, Zu-Hua Gao, Vincenzo Arena, Manuel Arena, Miguel N. Burnier, Goffredo Orazio Arena

**Affiliations:** 1 Cancer Research Program, McGill University Health Centre-Research Institute, 1001 Decarie Boulevard, Montreal, Quebec H4A 3J1 Canada; 2 Department of Pathology, McGill University Health Centre-Research Institute, 1001 Decarie Boulevard, Montreal, Quebec H4A 3J1 Canada; 3 Department of Obstetrics and Gynecology, Santo Bambino Hospital, via Torre del Vescovo 4, Catania, Italy; 4 Department of Surgical Sciences, Organ Transplantation and Advances Technologies, University of Catania, via Santa Sofia 84, Catania, Italy; 5 Department of Surgery, McGill University, St. Mary Hospital, 3830 Lacombe Avenue, Montreal, Quebec H3T 1M5 Canada

## Abstract

We reported on the ability of immortalized or oncosuppressor-mutated cells (OMCs) to uptake circulating cancer-factors and give tumors when transplanted into mice. This led to the first biological based liquid biopsy test, which we called MATER-D platform. In the present study, we showed for the first time that a different type of OMCs (PTEN-deficient human epithelial MCF10A cells) turn malignant when exposed to cancer patient’s sera, confirming the concept that different cells with diverse oncosuppressor mutations can uptake cancer factors and be used in biological based liquid biopsy tests. Our observations were confirmed in a large variety of solid and haematological malignancies. This test was able to detect dysplasia and carcinomas *in situ* lesions in different organs and circulating factors in cancer patients years after the removal of their lesions. To our knowledge, this ability is unique and not shared by other liquid biopsy platforms. Immunohistochemistry analysis of the xenotransplants revealed identical patterns of differentiation regardless of the cancer type, showing that differentiation through horizontal transfer might be dependent on the nature of the target cells rather than the type of cancer factors. These data strengthen the notion that OMC-based liquid biopsy tests might be promising platforms for cancer screening.

## Introduction

Horizontal transfer of molecules, which include signal proteins, bioactive lipids, and genetic material, through microvesicles or exosomes (EVs) is a recognized method of intercellular communication implemented in numerous physiological and pathological processes^1–12^.

In the last few years, the role of EVs in cancer genesis has been subjected to intense study due to recent discoveries on their role in cancer development, progression and metastatic niche formation^13–17^. Recently our group has demonstrated that exosomes isolated from the sera of cancer patients are able to transfer malignant traits to immortalized cells such as HEK293 (Human Embryonic Kidney cells) and oncosuppressor-mutated cells (OMCs) such as *BRCA1*-mutated human fibroblasts. Cancer exosomes would trigger their transformation into cancer cells as proved by the cancer masses that the treated OMCs generate when transplanted into NOD-SCID mice^18–21^. More important, we demonstrated that *BRCA1*-mutated fibroblasts turned into cancer cells, which exhibited phenotypical and immunohistochemical differentiation comparable to the cancer of the donor patients^18,19^. These discoveries, for the first time, confirmed the legitimacy of the hypothesis that the metastatic process might be secondary to an horizontal transfer of malignant traits rather than to circulating cells. According to this theory, metastatic disease might be independent from cell relocation and be a process reproducible at distance through the uptake of circulating cancer factors by primed cells located in target organs and their subsequent transformation into cancer cells with phenotypes analogous to the donor primary cancer cell^22–25^.

The ability of the OMCs to “sense” neoplastic factors circulating in the serum, uptake them, turn into malignant cells and give cancer masses when transplanted into NOD-SCID mice was further investigated by our group to verify if this peculiar feature could be incorporated into a biological platform and be used as a liquid biopsy test for cancer screening. This first biological based cancer-screening liquid biopsy test was called MATER-D platform (Metastatic and Transforming Elements Released Discovery platform) and preliminary evidence of its effectiveness in differentiating benign from malignant lesions and in detecting solid and hematological neoplasias was reported in a pilot study^26^.

In the present work, we aimed to verify if a different type of OMCs such as the human epithelial breast cell line MCF10A^27,28^ with a *PTEN* deletion was as effective in sensing oncogenic factors circulating in the sera of cancer patients as the OMCs we previously studied^18,19^.

The tumor suppressor gene *PTEN* (Phosphatase and tensin homolog) is a central negative regulator of the PI3K/AKT signaling cascade that influences multiple cellular functions including cell growth, survival, proliferation and migration in a context-dependent manner. Loss of PTEN function (due to mutations, deletions, or epigenetic silencing) is involved in many solid and hematological human malignancies^29,30^.

In addition to the above objective, we sought to strengthen the validity of the MATER-D platform and assess its effectiveness in detecting precancerous and early cancer lesions such as dysplasia and carcinoma *in situ* arising in different organs.

When exposed to cancer patient’s sera, MCF10A cells with a deletion of the *PTEN* gene turned malignant, as confirmed by the cancer masses obtained in NOD-SCID mice after xenotransplantation. *PTEN*-deficient MCF10A cells exposed to sera of a cohort of healthy patients, used as a control group, never gave any cancer masses when transplanted in NOD-SCID mice. These findings confirmed the concept that different types of cells with different oncosuppressor mutations can uptake circulating cancer factors, turn malignant and may be used in biological based liquid biopsy tests.

The MATER-D platform was able to detect dysplasia and carcinomas *in situ* lesions in the five different organs that were investigated (pancreas, colon, gallbladder, breast and skin). Surprisingly enough, circulating factors were detected in patients even years after the removal of precancerous and non-invasive lesions strengthening the validity of the hypothesis that the metastatic process might occur before cancer cells invasion of the basal membrane and be independent from cell migration.

Immunohistochemistry analysis of the xenotransplants obtained in mice after injection of OMCs treated with different cancer sera revealed that the immunohistochemistry patterns of malignant differentiation in *BRCA1*-mutated fibroblasts and *PTEN*-deficient MCF10A cells were always identical regardless of the cancer sera type that the cells were exposed to, confirming that horizontal transfer of malignant traits is a concept applicable also to epithelial cells (MCF10A) with a different oncosuppressor mutations (PTEN) than the ones previously investigated (BRCA1, P16 and P53)^19,20,25^. Furthermore, this unique discovery showed that malignant differentiation of target cells located in different organs, through horizontal transfer of oncofactors might be dependent on the histology of the target cells rather than the type of circulating cancer messengers.

Taken together, these observations shed new insights on the mechanisms behind the horizontal transfer of malignant traits and corroborate the validity of the claim that oncosuppressor-mutated cell-based liquid biopsy tests might be regarded as promising platforms for cancer screening, which deserve further study in properly designed clinical trials.

## Materials and Methods

### Patients’ recruitment and characteristics of cancers

Patients for the current study were recruited from the department of General Surgery at the Royal Victoria Hospital and St-Mary’s Hospital (Montreal, Canada) and underwent an informed consent for study participation (blood collection) in accordance to a protocol approved by the Ethics Committee of our institution (SDR-10-057). Blood samples were collected from four different cohorts of patients: healthy individuals (8 patients; C1−C8), patients with metastatic disease (5 patients; M1−M5), patients with clinical suspicion of cancer (12 patients; S1−S12) and patients admitted for treatment of different cancers or monitored for metastatic recurrence (6 patients; F1−F6) (Table 1). The inclusion criteria to be enrolled in the healthy cohort were: (i) age (30–60 year old), (ii) no signs and symptoms or personal history of cancer and (iii) family history negative for cancer.

**Table 1:** Clinical features of patients recruited in the present study.

Target	Case	Blood	Disease	Transfo	Met.	Age	Sex	Tumor size
Cells	#	Collection^b^		Pot.		(year)		+/− SD (cm^3^)^†^
Fibro-	C1		Healthy	No				0.000 +/− 0.000
BKO	C2		Healthy	No				0.000 +/− 0.000
	C3		Healthy	No				0.000 +/− 0.000
	C4		Healthy	No				0.000 +/− 0.000
	S1	Pre-op	Gallbladder High grade dysplasia	Yes	No	80	Male	0.050 +/− 0.016*
	S1.1	Post-op (2 years)	Gallbladder High grade dysplasia	Yes	No	81	Male	0.032 +/− 0.004*
	S2	Pre-op	Bile duct lesion	Yes	No		Male	0.136 +/− 0.016*
	S3	Pre-op	Colonic Polyp	No	No	76	Male	0.000 +/− 0.000
	S4	Pre-op	Colonic polyp	No	No	62	Male	0.000 +/− 0.000
	S5	Pre-op	Pancreatic Cyst *in situ*	Yes	No		Male	0.045 +/− 0.007*
	S6	Pre-op	Melanoma	Yes	No	54	Male	0.184 +/− 0.030*
	S7	Post-op (5 years)	Dysplastic Melanocytic Nevus	Yes	No	47	Female	0.022 +/− 0.002*
	S8	Pre-op	Breast cancer (DCIS)	Yes	No	52	Female	0.272 +/− 0.003*
	S9^a^	Pre-op	Breast cancer (Invasive carcinoma)	Yes	No		Female	0.916+/− 0.105*
	S10	Pre-op	Thyroid nodule	No	No		Female	0.000 +/− 0.000
	S11	Pre-op	Colonic Polyp (high grade dysplasia)	Yes	No		Female	0.053+/− 0.011*
	F1^a^	Post-op (visit 2; 1 year)	Lung Cancer	Yes	No	78	Female	0.147 +/− 0.067*
	F1.1	Post-op (visit 3; 2 years)	Lung Cancer	Yes	Yes	79	Female	0.708 +/− 0.071*
	F1.2	Post-op (visit 4; 3 years)	Lung Cancer	Yes	Yes	79	Female	0.262 +/− 0.021*
	F2	Post-op (5 years)	Pancreatic, Colon, Prostate	Yes	No		Male	0.510 +/− 0.015*
	F3^a^	Post-op (6 years)	Lymphoma + GIST	Yes	No	73	Female	0.252 +/− 0.006*
	F4^a^	Post-op (4 years)	Lymphoma	Yes	No	68	Female	0.213 +/− 0.042*
	F5	Post-op (8 years)	Choroidal Melanoma	Yes	No	61	Male	0.114 +/− 0.019*
	F6	Post-op (10 years)	Colonic Polyp; Carcinoma *in situ*	Yes	No	75	Male	0.001 +/− 0.000*
MCF-	C5		Healthy	No				0.000 +/− 0.000
PKO	C6		Healthy	No				0.000 +/− 0.000
	C7		Healthy	No				0.000 +/− 0.000
	C8		Healthy	No				0.000 +/− 0.000
	M1		HCC	Yes	Yes	61	Female	0.035 +/− 0.002*
	M2		CRCLM	Yes	Yes	66	Female	0.033 +/− 0.002*
	M3		Ovarian cancer-LM	Yes	Yes	58	Female	0.027 +/− 0.003*
	M4		CRCLM	Yes	Yes			0.033 +/− 0.002*
	M5		Melanoma	Yes	Yes			0.027 +/− 0.002*
	S12	Pre-op	Pancreatic cancer-LM	Yes	Yes		Male	0.019 +/− 0.002*
	S9^a^	Pre-op	Breast cancer (Invasive carcinoma)	Yes	No		Female	0.057+/− 0.018*
	F1^a^	Post-op (visit 2; 1 year)	Lung Cancer	Yes	No	78	Female	0.072 +/− 0.009*
	F3^a^	Post-op (6 years)	Lymphoma + GIST	Yes	No	73	Female	0.030 +/− 0.001*
	F4^a^	Post-op (4 years)	Lymphoma	Yes	No	68	Female	0.025 +/− 0.003*

CRCLM: ColoRectal Cancer-Liver Metastasis, HCC: Hepatocellular Carcinoma, DCIS: ductal cancer *in situ*, GIST: Gastrointestinal Stromal Tumor.

Fibro-BKO: *BRCA1*-mutated fibroblasts, MCF-PKO: *PTEN*-mutated MCF10A.

Transfo. Pot.: transformation potential of sera as assessed following cell exposure and subcutaneous transplantation in NOD/SCID mice.

Met.: metastatic status as assessed during clinical monitoring.

^a^These samples were tested on both cell types.

^b^In brackets; blood collection after primary tumor resection.

^†^Tumor sizes were measured at mice euthanasia and were expressed as mean +/− SD (n = 4–6 xenotransplants per samples), *P < 0.05 as compared to the respective control groups.

C: Healthy Control; S: Screening; F: Follow-up; M: Metastasis.

### Blood collection and serum preparation from cancer patients and healthy subjects

Blood samples were collected from a peripheral vein in vacutainer tubes (Becton Dickinson) containing clot-activation additive and a barrier gel to isolate serum. Blood samples were incubated for 60 min at room temperature to allow clotting and subsequently were centrifuged at 1500 g for 15 min. Serum was collected and a second centrifugation was performed on the serum at 2000 g for 10 min, to clear it from any contaminating cells. Serum samples were aliquoted and stored at −80 °C until use.

### Cell lines and culture conditions

We used the CRISPR/Cas9 system to establish a stable *BRCA1*-KO in human fibroblasts (Fibro-BKO) as previously described^19^. *PTEN*-deficient MCF10A cells (MCF-PKO: MCF10A PTEN−/−; 10A5Pc1) were supplied by Dr Kurtis Bachman^31^. Cells were maintained as per supplier’s recommendations in complete DMEM-F12 medium (i.e. DMEM-F12 medium supplemented with 5% horse serum, 0.5 μg/ml hydrocortisone, 100 ng/ml cholera toxin, 10 μg/ml insulin, 20 ng/ml hEGF, and penicillin/streptomycin antibiotics). When cells reached 30% confluence, they were treated with complete DMEM-F12 medium (Wisent, Saint-Bruno, Canada) supplemented with 10% cancer patient sera or control sera, which had been filtered through 0.2 μm filters. Cells were maintained in patient sera-supplemented complete medium (cancer and healthy control) at 37 °C in humidified atmosphere containing 95% air and 5% CO_2_ with medium change every second day for the whole 3 weeks. When cells reached 80–90% confluence, they were passaged 1 in 6 using 0.05% Trypsin-EDTA (Wisent, Saint-Bruno, Canada). To confirm that there was no contamination or carry-over of cells from human serum, aliquots of the culture medium were placed in a culture plate and incubated at 37 °C, 5% CO_2_ for 4 weeks.

### Immunoblotting

Cells were lysed in RIPA buffer containing protease inhibitors (Sigma, Oakville, Canada). Equal amounts of proteins were resolved on 10% SDS-PAGE and transferred to a nitrocellulose membrane (BioRad, CA, USA). Membranes were blocked in TBS containing 5% non-fat dry milk and exposed overnight at 4 °C to rabbit-anti-PTEN (ab154812, Abcam, MA, USA) or mouse-anti-β-Actin (A5316, Sigma, Oakville, Canada) antibodies. Membranes were washed in TBST (TBS-0.05% Tween-20) and incubated with either anti-rabbit or anti-mouse peroxidase-conjugated secondary antibody for 1 hour at room temperature and the blots were developed using Immobilon Western HRP Substrate (Millipore, Etobicoke, Canada).

### *In vivo* tumor growth

Five-week-old female NOD-SCID mice (Jackson Laboratory) were used with approval and in compliance with McGill University Health Centre Animal Compliance Office (Protocol 2012–7280). Cells growing in log phase were harvested by trypsinization and washed twice with HBSS. Mice (2 to 3 mice) were injected subcutaneously in the right flank with 2 million cells in 200 μl HBSS/Matrigel mixture. The resulting xenotransplants were photographed and processed for immunohistochemistry.

### Immunohistochemistry labelling procedures and histological analyses

Mice xenotransplants were collected, fixed in 10% buffered formalin, embedded in paraffin, stained with H&E (hematoxylin and eosin) according to standard protocols and processed for immunohistochemistry. Briefly, 5 μm tissue sections were dewaxed in xylene and rehydrated with distilled water. After antigen unmasking, and blocking of endogenous peroxidase (3% hydrogen peroxide), the slides were incubated with primary antibodies (Supplementary Table S1). Labeling was performed using iView DAB Detection Kit (Ventana) on the Ventana automated immunostainer. Sections were counterstained lightly with Hematoxylin before mounting. A certified pathologist who was blinded to the clinical characteristics of the cancer patients, whose sera were being analyzed, performed the histological analyses. The intensity of membrane staining for CK7, CK19, CK20, AE1/AE3, CK34BE12, CD45 and HMB45, cytoplasmic staining for mammoglobin, hep par-1, vimentin, and nuclear staining for CDX2, TTF1 and P63 were graded blindly by the pathologist and scored using a 4-tiered (0–3) grading system as previously described^32^.

### Statistical analysis

Statistical differences were analyzed using an ANOVA followed by the Dunnett test for multiple comparisons with one control group. A p value < 0.05 was considered statistically significant.

### Ethics approval and consent to participate

Patients recruited for this study underwent an informed and written consent for blood collection in accordance to a protocol approved by the Ethics Committee of the McGill University Health Centre (Reference. SDR-10-057). Mice involved in this study were used with the approval and in compliance with the McGill University Health Centre Animal Compliance Office (Protocol 2012–7280).

## Results

### *PTEN*-deficient MCF10A cells underwent malignant transformation when exposed to sera of cancer patients

**Figure 1:**
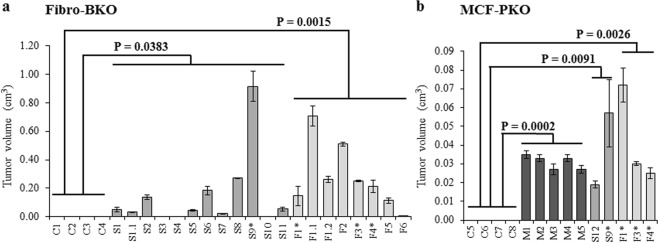
Xenotransplants obtained following treatment with different cancer patients’ sera. *BRCA1*-mutated fibroblasts (Fibro-BKO (**a**)) and *PTEN*-deficient MCF10A cells (MCF-PKO (**b**)) were cultured for 3 weeks in sera obtained from healthy donors or cancer patients. Treated cells were injected subcutaneously in NOD-SCID mice. Four weeks after injection, xenografts volumes were calculated. Values are mean ± SD, (n = 4–6 xenotransplants per treatment). P values refer to the statistics between the different treated groups and the control group as follows: (**a**) Control vs. Screening (P = 0.0383), and control vs. Follow-up (P = 0.0015), (**b**) Control vs Metastatic (P = 0.0002), Control vs. Screening (P = 0.0091), and control vs. Follow-up (P = 0.0026).

We reported that mutated human fibroblasts and immortalized embryonic kidney cells were prone to malignant transformation when exposed to sera of cancer patients^18–20^. In the present study, we sought to verify if this behavior was also shared by epithelial cells carrying a different mutation than the previously mutations examined (Table 1, Fig. 1). *PTEN-*deficient MCF10A cells, a human mammary epithelial cell line, that has not shown to be tumorigenic (herein called MCF-PKO cells)^27,28,31,33^ was treated with sera of cancer patients and its hypothesized malignant transformation was compared to the transformation obtained in a positive control group made of *BRCA1*-mutated fibroblasts (herein called Fibro-BKO cells) treated mostly with the same type of cancer sera. MCF10A cells deficient for *PTEN* were generated by a biallelic deletion of the *PTEN* gene. *PTEN* deletion was confirmed by Western blot that showed a complete absence of PTEN expression (Supplementary Fig. S1). We tested the blood of four healthy individuals (Cases C5–C8) and five patients who were to undergo resection of primary cancer or were readmitted for treatment of metastatic disease (Cases M1–M5) (Table 1). At the end of the *in vitro* treatments, cells were injected subcutaneously in NOD-SCID mice, tumor growth was monitored, and tumor volumes were calculated at euthanasia (Table 1, Fig. 1).

MCF-PKO cells treated with sera of cancer patients underwent malignant transformation as confirmed by tumor formation following transplantation in NOD-SCID mice (Fig. 1b). In contrast, none of the mice injected with MCF-PKO cells cultured with healthy control sera developed visible tumors (Fig. 1b). To demonstrate that *PTEN* deletion in the MCF10A cells is a prerequisite to elicit malignant transformation in response to cancer patient serum, maternal MCF10A cells were treated with healthy control (n = 3) or cancer patient sera (n = 3 CRCLM sera) (Supplementary Fig. S2). None of the mice injected with these cells developed tumors during the course of the experiments (4 weeks latency). The matrigel plug excised from the site of injection did not show the presence of any tumor epithelium. H&E staining showed only fibrous tissue with ghost cells (Supplementary Fig. S2). These findings corroborate the evidence, already documented in previous publications from our group, that any oncosuppressor-mutated cell (i.e. mesenchymal, epithelial, embryonic or adult) is capable to sense cancer-derived circulating oncogenic factors, uptake them and turn malignant, regardless of the induced or acquired oncosuppressor mutation. This new evidence of the receptivity of OMCs to the horizontal transfer of malignant traits strengthens the notion that such cells can be used as biological indicators of circulating cancer factors.

### MATER-D platform detected circulating transforming factors in patients with dysplastic lesions and *in situ* carcinomas

In order to assess the effectiveness of the OMCs in detecting circulating cancer factors even at the early neoplastic stages such as dysplasia and *in situ* carcinoma, we recruited eleven patients with clinical suspicion of cancer in different organs (skin, colon, breast, gallbladder, thyroid and pancreas) and normal tumor markers. We enrolled these patients in the screening group and we tested them with the MATER-D platform. Eleven patients’ sera were tested using Fibro-BKO cells as biological platform and two patients sera were tested using MCF-PKO cells. One of those patients was tested with both cell lines (Case 9) (Table 1). All patients then underwent surgery for the excision of the suspicious lesions and the results of the pathology were compared with the results obtained on the MATER-D platform. Nine out of the twelve patients that were screened transformed the OMCs into cancerous cells and therefore tested positive on the MATER-D platform. Three patients (Cases S3, S4 and S10) tested negative since no cancerous transformation of the OMCs occurred.

When the results from the pathology analysis were checked against the results of the MATER-D test, it was found that the MATER-D test had resulted positive not only in patients with fully developed cancer but also in patients with high grade dysplasia or carcinomas *in situ* (Table 1). The three patients (Cases S3, S4 and S10) that tested negative on the MATER-D platform, on pathology were found to have benign colonic polyps only (Cases S3 and S4) and benign thyroid nodule (Case S10), with no signs of high-grade dysplasia or *in situ* invasiveness. Out of eight cases, the MATER-D platform was sensitive enough to detect the presence of dysplasia (n = 5) and distinguish benign lesions (n = 3) from malignant tumors. These findings suggest that the MATER-D platform is effective in detecting circulating neoplastic factors even before the full neoplastic transformation and in the absence of tumor markers positivity. These data altogether indicate that OMCs could be used as a liquid biopsy tests for early cancer screening.

We also used the MATER-D platform to analyze the sera of five patients that were seen in follow-up after resection of dysplastic lesions or primary tumor. Our aim was to verify if circulating cancer factors were still present after cancer resection and be theoretically responsible for cancer recurrence in other organs (Table 1). Three patient’s sera were tested on Fibro-BKO only (Cases F2, F5 and F6) and three patients were tested on both cell lines (Cases F1, F3 and F4). The sera of all six patients still tested positive and were still retaining their transforming abilities even years after primary tumor resection (Table 1, Fig. 1). These data suggest that neoplastic factors are still circulating in the serum of cancer patients even after curative resection has been performed and give evidence that horizontal transfer of malignant traits can still occurs even years after the primary tumor has been excised. Furthermore, the presence of circulating cancer factors in patients with dysplastic lesions even after excision of the lesions suggests that cell invasion of the basal membrane might not be the only mechanism behind local or distant cancer recurrence. These results strongly indicate that the model of horizontal transfer of malignant traits should be further evaluated as an alternative mechanism to explain both synchronous and metachronous metastatic disease.

### Oncosuppressor-mutated cells follow the same pattern of differentiation and express the same immunohistochemistry markers regardless of the type of cancer sera they are exposed to

**Figure 2:**
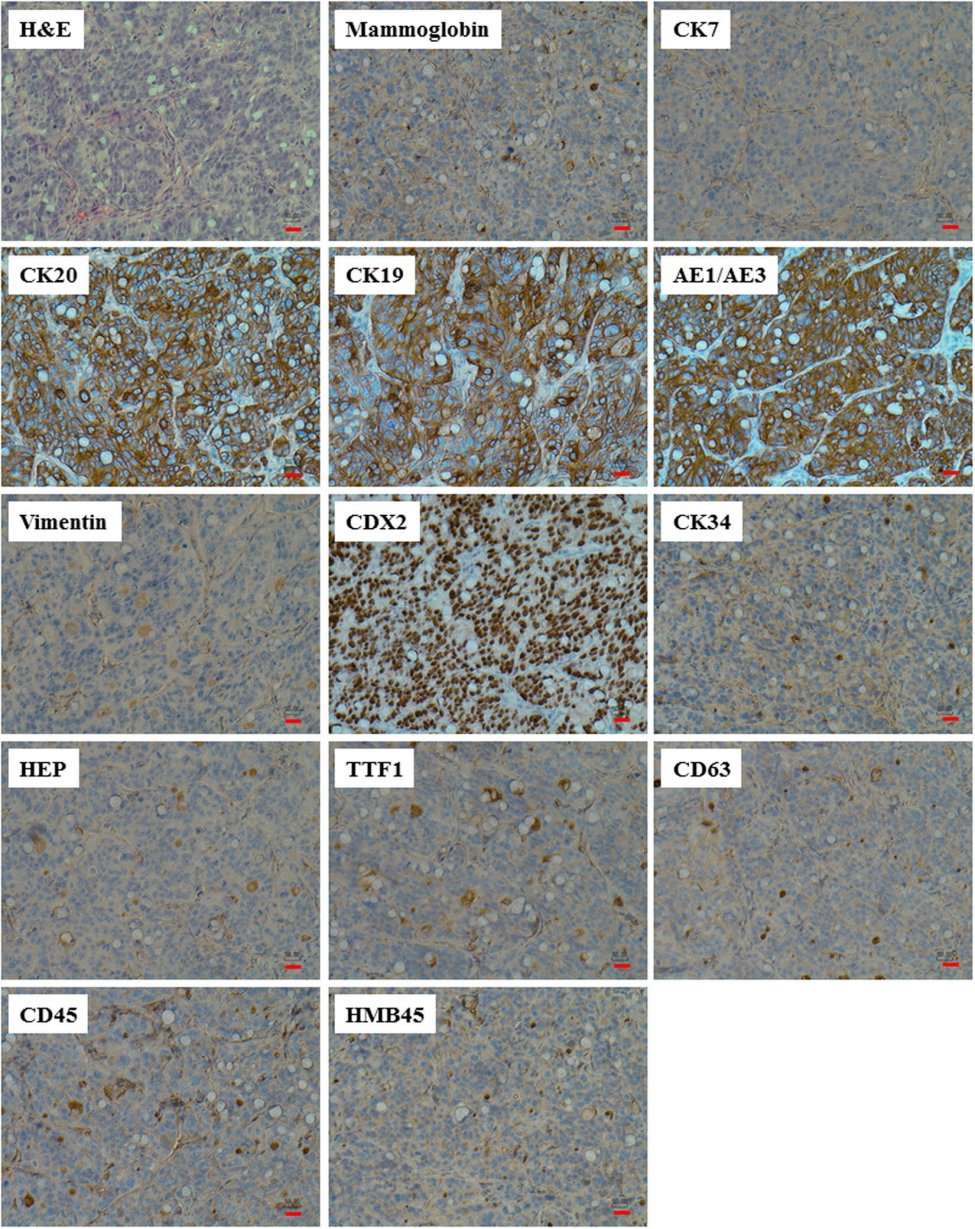
*BRCA1*-mutated fibroblasts express the same phenotypical markers regardless of the type of cancer sera they are exposed to. *BRCA1*-KO fibroblasts were treated with cancer patient sera for 3 weeks. Treated cells were injected subcutaneously into NOD**/**SCID mice that were followed for 4 weeks for tumors growth. Developing tumors were excised, fixed and embedded in paraffin. Xenotransplant samples were processed for H&E staining, and immunolabeled with antibodies to different cancer markers. Representative pictures shown are from cells exposed to case S8. Scale bars: 50 µm.

**Figure 3:**
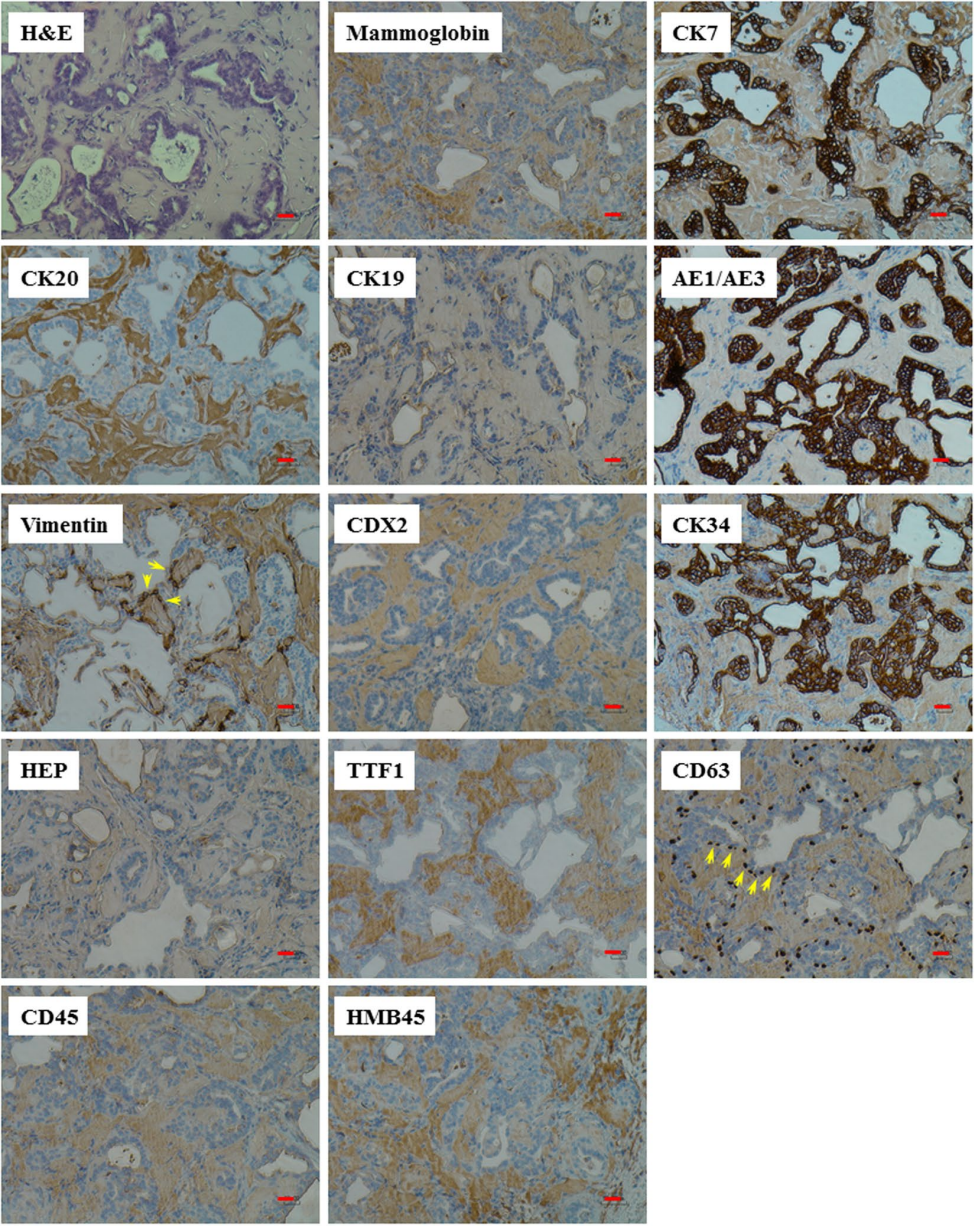
*PTEN*-deficient MCF10A cells express the same phenotypical markers regardless of the type of cancer sera they are exposed to. *PTEN*-KO cells were treated with cancer patient sera for 3 weeks. Treated cells were injected subcutaneously into NOD**/**SCID mice that were followed for 4 weeks for tumors growth. Developing tumors were excised, fixed and embedded in paraffin. Xenotransplant samples were processed for H&E staining, and immunolabeled with antibodies to different cancer markers. Representative pictures shown are from cells exposed to case M5. Scale bars: 50 µm. Yellow arrows point to basal cells.

Recently our group, for the first time, published novel evidence that Fibro-BKO cells exposed to exosomes extracted from patients with colo-rectal cancer, hepatocellular carcinoma, pancreatic cancer and biliary tract cancers would turn into cancer cells whose phenotype and immunohistochemistry analysis was matching the cancer of origin^18,19^. To verify if the MCF-PKO cells had the same capability displayed by Fibro-BKO cells and to assess the extent of their transforming potential, tumors formed from xenotransplants of both Fibro-BKO and MCF-PKO cells were analyzed by an independent pathologist who was blinded to the clinical characteristics of the cancer patients whose sera was used to transform the OMCs. To better analyze the range of malignant transformation achieved by the OMCs, the pathologist performed the same immunohistochemistry panel that is commonly used in cases of cancers of unknown origin (Supplementary Table S1), and stains for the following markers were completed for all twenty patients: CK7, CK20, AE1AE3, CK19, vimentin, CDX2, CK34BE12, Hep-Par1, TTF1, P63, CD45, HMB45 (Tables 2 and 3, Figs 2 and 3). Independently of the cancer serum used, excised xenotransplants displayed features of adenocarcinomas (H&E staining) with high proliferation index for tumor arising from Fibro-BKO cells (80–93% Ki67 positivity), and moderate proliferation index for tumor arising from MCF-PKO cells (20–45% Ki67 positive cells). Fibro-BKO cells exposed to cancer sera completely changed their fate since all excised tumors stained negative for vimentin, which is normally expressed on fibroblasts (Table 2, Fig. 2)^34^. Moreover, Fibro-BKO cells displayed a pattern of markers expression suggestive of lower gastrointestinal tract differentiation (CK7^−^/CK20^+^/CDX2^+^) regardless of the cancer sera used (Table 2, Fig. 2). MCF-PKO cells also displayed the same pattern of differentiation in all cases (Table 3, Fig. 3). Notably, the phenotypical pattern of the MCF-PKO cells was consistently suggestive of upper gastrointestinal tract differentiation (CK7^+^/CK20^−^/CDX2^−^) (Table 3, Fig. 3). To verify whether the different pattern of differentiation was secondary to the circulating oncofactors or to the histology of the uptaking cell, Fibro-BKO cells and MCF-PKO cells were exposed to the sera of the same patient in three different cancer cases (breast cancer, lymphoma and lung cancer) (Tables 2 and 3). Surprisingly enough, the Fibro-BKO cells were always CK7 negative, CK20 positive and CDX2 positive and the MCF-PKO cells were always CK7 positive, CK20 negative and CDX2 negative despite being exposed to the serum of the same patient and therefore to the same circulating cancer factors. The pattern of differentiation didn’t change even when sera from different cancers were used (breast cancer, lymphoma and lung cancer) (Tables 2 and 3). Altogether, these results suggest that the horizontal transfer of malignant traits might involve different types of cells and indicate that the patterns of differentiation might be limited by the histology of the uptaking cells. The circulating oncofactors if uptaken by the cells would prompt a cascade of events that would lead the cells to malignant transformation, and differentiation with expression of a stable set of cytokeratin markers.

**Table 2:** Immunohistochemical profile of *BRCA1*-deficient fibroblast xenotransplants.

Cases	Mammo	CK7	CK20	CK19	AE1/AE3	Vimentin	CDX2	CK34BE12	Hep-Par1	TTF1	P63	CD45	HMB45
S1	−	Rare + vity	3+	3+	3+	−	3+	−	−	−	−	−	−
S1.1	ND	ND	ND	ND	ND	ND	ND	ND	ND	ND	ND	ND	ND
S2	−	−	3+	2+	3+	−	3+	−	−	−	−	−	−
S3	NT	NT	NT	NT	NT	NT	NT	NT	NT	NT	NT	NT	NT
S4	NT	NT	NT	NT	NT	NT	NT	NT	NT	NT	NT	NT	NT
S5	−	−	3+	3+	3+	−	3+	−	−	−	−	−	−
S6	−	−	3+	3+	3+	−	3+	−	−	−	−	−	−
S7	−	−	3+	3+	3+	−	3+	−	−	−	−	−	10% + ve cells
S8	−	−	3+	3+	3+	−	3+	−	−	−	−	−	−
S9*	−	−	3+	3+	3+	−	3+	−	−	−	−	−	−
S10	NT	NT	NT	NT	NT	NT	NT	NT	NT	NT	NT	NT	NT
S11	−	−	3+	2+	3+	−	3+	−	−	−	−	−	−
F1*	−	Rare + vity	3+	3+	3+	−	3+	−	−	−	−	−	−
F1.1	ND	ND	ND	ND	ND	ND	ND	ND	ND	ND	ND	ND	ND
F1.2	−	−	3+	3+	3+	−	3+	−	−	−	−	−	−
F2	−	−	3+	3+	3+	−	3+	−	−	−	−	−	−
F3*	−	−	3+	3+	3+	−	3+	−	−	−	−	−	−
F4*	−	−	3+	−	3+	−	3+	−	−	−	−	−	−
F5	−	−	3+	3+	3+	−	3+	−	−	−	−	−	−
F6	−	−	3+	3+	3+	−	3+	−	−	−	−	−	−

Mammo: Mammoglobine; −: Negative; +: Positive; NT: No Tumor; ND: Not Done. *Cases also run on *PTEN*-deficient MCF10A cells.

**Table 3:** Immunohistochemical profile of *PTEN*-deficient MCF10A xenotransplants.

Cases	Mammo	CK7	CK20	CK19	AE1/AE3	Vimentin	CDX2	CK34BE12	Hep-Par1	TTF1	P63	CD45	HMB45
M1	5% cell 2+	3+	−	−	3+	Basal cells 3+	−	3+	Focal 1+	−	Basal cells 3+	−	−
M2	−	2+	−	−	3+	Basal cells 3+	−	3+	−	−	Basal cells 3+	−	−
M3	−	3+	−	−	3+	Basal cells 3+	−	3+	−	−	Basal cells 3+	−	−
M4	−	3+	−	−	3+	Basal cells 3+	−	3+	−	−	Basal cells 3+	−	−
M5	−	3+	−	−	3+	Basal cells 3+	−	3+	−	−	Basal cells 3+	−	−
S12	−	3+	−	−	3+	Basal cells 3+	−	3+	−	−	Basal cells 3+	−	−
S9*	−	3+	−	−	3+	Basal cells 3+	−	3+	−	−	Basal cells 3+	−	−
F1*	−	3+	−	−	3+	Basal cells 3+	−	3+	−	−	Basal cells 3+	−	−
F3*	−	3+	−	−	3+	Basal cells 3+	−	3+	−	−	Basal cells 3+	−	−
F4*	−	3+	−	3+	3+	Basal cells 3+	3+	3+	−	−	Basal cells 3+	−	−

Mammo: Mammoglobine; −: Negative; +: Positive. *Cases  also run on *BRCA1*-deficient fibroblasts.

## Discussion

In the present study we show the latest findings observed while studying the horizontal transfer of malignant traits to oncosuppressor mutated cells and we report new data on the effectiveness of the MATERD platform as a biological liquid biopsy.

We showed that epithelial cells with a deletion of the oncosuppressor gene *PTEN* turn malignant, when exposed to sera of patients with cancer, confirming the evidence that even epithelial cells, carrying an oncosuppressor mutation, possess the ability to uptake cancer derived factors and be susceptible of malignant transformation^18,19^. To our knowledge, this is the first time that evidence is brought about on the possibility that the horizontal transfer of malignant traits might also involve epithelial cells and evidence is generated that the oncosuppressor gene *PTEN* might be another gene involved in this phenomenon as it was already shown with the oncosuppressor genes *BRCA1*, P16 and P53^19,20,25^.

When we elected to test the MCF10A oncosuppressor knocked-out cell line, our intention was threefold: to extend the validity of the concept of horizontal transfer of malignant traits to epithelial cells, to assess their range of malignant differentiation and verify if we could achieve patterns of phenotypical differentiation that we were not capable to reproduce in fibroblasts carrying a *BRCA1* mutation. The results herein reported, of a constant pattern of differentiation that the Fibro-BKO and MCF-PKO show, when exposed to different cancer sera, is quite stupefying and intriguing. Since it is the first time that an attempt to study the range of immunohistochemical differentiation of two different OMCs with two different oncosuppressor mutations is done, no definitive conclusions can be drawn. Nevertheless, the peculiarity of these results certainly justifies the legitimacy of daring scientific speculations and thought stimulating hypotheses. The evidence that Fibro-BKO cells always displayed a pattern of markers expression suggestive of lower gastrointestinal tract differentiation (CK7^−^/CK20^+^/CDX2^+^) and the confirmation that the phenotypical pattern of the MCF-PKO cells was consistently suggestive of upper gastrointestinal tract differentiation (CK7^+^/CK20^−^/CDX2^−^)^35^ prompt us to speculate that if metastases are secondary to circulating factors rather than circulating cells, the target cells that uptake the onco-information and turn malignant would vary depending on the type of the cancer that releases the oncofactors. In other words, cancers arising from the lower gastrointestinal tract would release cancer factors that would be uptaken preferentially by fibroblasts located in target organs as opposed to cancer factors released by tumors arising from the upper gastrointestinal tract that would be mainly uptaken by epithelial cells located in distant organs. For example, colo-rectal cancer factors (lower gastrointestinal tract) would preferentially “metastasize” to fibroblasts, whereas pancreatic cancer transforming factors (upper gastrointestinal tract) would mainly “metastasize” to epithelial cells contained in distant organs such as liver, lung and bones. The fibroblasts that uptake the malignant traits released by colon cancer cells would express the immunohistochemistry markers that are typical of cancers from the lower GI tract mimicking colon cancer cells whereas epithelial cells would preferentially uptake pancreatic cancer factors, turn malignant, express the pattern typical of upper gastrointestinal tract differentiation and mimic a pancreatic cancer cell on pathology examination. In line with this way of thinking, it can be hypothesized that patients, who develop a second type of cancer, might not actually have a new type of cancer. In reality the new cancer would be consequence of the same oncofactors inducing malignant transformation in a different uptaking cell. For example, oncofactors released by colon cancer cells would give metachronous metastases if uptaken and expressed by fibroblasts in the liver (CK7^−^/CK20^+^/CDX2^+^) or a second type of cancer such as a cholangiocarcinoma (upper gastrointestinal tract and CK7^+^/CK20^−^/CDX2^−^) if they were uptaken and expressed by epithelial liver cells^22,35^. The clinical evidence that second primary cancers usually express a CK7/CK20 pattern opposite to the first primary seems to justify the legitimacy of this hypothesis^36,37^. It must be mentioned that the *in vitro* transformation of BRCA1 KO fibroblasts is not supported by evidence that synchronous or metachronous metastases can occur in fibroblasts, *in vivo*. Therefore, although this hypothesis seems enticing, this event might have no physiological relevance and be a phenomenon limited to *in vitro* settings.

Recently, we reported that cancer exosomes are enriched in integrins^18^. These proteins are associated with the preferential fusion of the exosomes in target tissues and it has already been suggested that the uptaking target cells might be determined by the type of receptors expressed on the membrane and their specific interaction with specific cancer exosomes-expressed integrins^38^.

Whether the different phenotype expression that we observed is a consequence of an intrinsic limitation of the cells to differentiate or is secondary to the type of oncosuppressor deficiency present in the cell (BRCA1 vs PTEN) can be a matter of debate and it is a hypothesis that mandates further investigation. This significant limitation of our study therefore prevents us from drawing any definitive conclusions. Nevertheless, the singularity of these findings, never reported before, and the awareness of their possible implications in the field of the horizontal transfer of oncological traits compel us to share them with the scientific community in the hope to stimulate interest and encourage constructive criticism that will certainly help to understand the mechanism behind this fascinating phenomenon.

The discovery of the capability of OMCs to uptake circulating cancer factors and turn malignant not only strengthened the validity of the horizontal transfer of malignant traits as an alternative model to explain metastatic disease, but was also used by our group to design the first biological based cancer-screening liquid biopsy test, which we called MATER-D platform^26^. All the liquid biopsies being investigated or currently in use are based on the biochemical detection of somatic mutations that are expected to be found only in malignant cells circulating in patients affected by cancer disease^39–44^.

The detection of circulating tumor DNA and its genotyping is achieved through several methods including the application of next generation sequencing together with advanced computational methods^43^. Although the sensitivity of liquid biopsies has improved in the last few years unfortunately its diagnostic sensitivity in patients with early-stage cancer has not progressed accordingly^43,44^.

The MATER-D platform exploits the capability of OMCs to sense and uptake those circulating malignant factors that are able to turn the OMCs into cancer cells and are suspected to have a biological effect on cells *in vivo*. By recreating *in vitro* a biological environment close to the physiological background in which cancer genesis occurs, the sensitivity of the MATER-D platform might prove to be superior to other liquid biopsies, as demonstrated by the ability to detect not only carcinoma *in situ* but also high-grade dysplastic lesions with 100% accuracy. Furthermore, the negative results obtained on patients that turned out on pathology to have tubular adenoma of the colon with no malignant potential or benign thyroid cysts, strengthen our assumption that biological based platforms hold promising potential for cancer screening due to the likely ability to distinguish between a benign process and a potentially malignant lesion.

It is our belief that a proper understanding of the mechanisms that drive the OMCs to uptake circulating cancer messengers and turn cancerous might also improve the sensitivity of the liquid biopsies based on cancer gene sequencing. As a matter of fact, the identification of the specific circulating factors, responsible for the malignant transformation of the OMCs, might help narrow the number of genes that needs to be targeted and sequenced in conventional liquid biopsies and increase their precision and sensitivity.

The discovery that cancer factors might be present in the sera of patients even years after resection of dysplastic lesions raises important questions and suggests several provocative hypotheses. Dysplastic lesions, by definition, are made of cells that have not invaded the basal membrane and therefore are not cancerous yet, since one of the hallmarks of cancer is invasion with subsequent ability to metastasize. The fact that OMCs turn malignant when exposed to sera of patients with dysplastic lesions indicate that cancer factors circulate in the blood before the full neoplastic transformation is complete and cell invasion has occurred, and suggest that oncofactors are present in the blood even after localized lesions, with no invasive features, have been resected. These findings certainly do not fit in the conventional model and somewhat shake the foundations of the mainstream theory. These results raise the suspicion that metastases might not need cell invasion in order to occur and foster the hypothesis that cancer might be a chronic systemic disorder even at the early stages^22^. Further experiments are needed to confirm the appropriateness of these hypotheses since the results herein reported necessitate to be validated and confirmed in a much larger number of patients enrolled in a properly designed clinical trial.

A limitation of the MATER-D platform, which is also shared with other liquid biopsies, is the inability of the test to localize the exact location of the neoplastic process. The patients that were screened and were found to be positive still had to undergo a battery of radiological and endoscopic exams to localize the lesion producing the circulating factors. Another important weakness, which is intrinsic to this biological platform, is the requirement of mice in order to assess the malignant transformation of the treated OMCs. This condition prevents the widespread utilization of this method since animal facilities are not normally available in common diagnostic laboratories. To overcome this problem we are testing some new biological platforms that don’t necessitate mice xenotransplantation to confirm the results of the screening and will make the screening process faster and easier to perform.

## Conclusion

The horizontal transfer of malignant traits to OMCs is a fascinating phenomenon that warrants further study in order to understand its value in cancer genesis and more important its role in the metastatic process. The discovery that MCF-PKO cells with a PTEN deletion undergo malignant transformation confirms that the horizontal transfer of malignant traits is a phenomenon that is not limited to a type of cell or a specific oncosuppressor gene mutation but it is a proven and reproducible behavior, which might have an important role in understanding cancer disease. Biological based liquid biopsies such as MATER-D hold promising potentials that necessitate further testing in clinical trials to confirm their efficacy and sensitivity. The evidence that dysplastic lesions release cancer factors in the blood and their presence in the serum of patients years after their surgical resection seems to challenge the principles of the current dogma. These results need further rigorous testing and severe scrutiny for their possible implications in the future of cancer research.

## Supplementary information

Supplementary Information

## Data Availability

All data generated and analyzed during this study are included in this manuscript and in its supplementary information files.
