# Supplementary material for: Oncosuppressor-Mutated Cells as a Liquid Biopsy Test for Cancer-Screening
Source: Sci Rep. 2019 Feb 20;9:2384. doi: 10.1038/s41598-019-38736-y (PMC6382857; doi:10.1038/s41598-019-38736-y)
Supplement: Supplementary file 1 — Supplementary Information [file 41598_2019_38736_MOESM1_ESM.pdf]

Oncosuppressor-Mutated Cells as a Liquid Biopsy Test for Cancer-Screening.

Mohamed Abdouh[1], Zu-Hua Gao[2], Vincenzo Arena[3], Manuel Arena[4], Miguel N. Burnier[1] & Goffredo Orazio Arena[1,5*]

[1] Cancer Research Program, McGill University Health Centre-Research Institute, 1001 Decarie Boulevard, Montreal, Quebec, Canada, H4A 3J1

[2] Department of Pathology, McGill University Health Centre-Research Institute, 1001 Decarie Boulevard, Montreal, Quebec, Canada, H4A 3J1

[3] Department of Obstetrics and Gynecology, Santo Bambino Hospital, via Torre del Vescovo 4, Catania, Italy

[4] Department of Surgical Sciences, Organ Transplantation and Advances Technologies, University of Catania, via Santa Sofia 84, Catania, Italy

[5] Department of Surgery, McGill University, St. Mary Hospital, 3830 Lacombe Avenue, Montreal, Quebec, Canada, H3T 1M5

Supplementary Table 1. List of antibodies used in this study.

Antibodies ID	Species	Manufacturer
Ki67	Rabbit Monoclonal	Ventana (USA)
AE1/AE3	Mouse Monoclonal	DAKO (Denmark)
CK7	Mouse Monoclonal	DAKO (Denmark)
CK20	Mouse Monoclonal	DAKO (Denmark)
CDX-2	Rabbit Monoclonal	Cell MARQUE (USA)
CK19	Mouse Monoclonal	DAKO (Denmark)
Vimentin	Mouse Monoclonal	Ventana (USA)
HEP-PAR1	Mouse Monoclonal	Ventana (USA)
Mammoglobine	Mouse & Rabbit Cocktail	Cell MARQUE (USA)
CK34BE12	Mouse Monoclonal	DAKO (Denmark)
TTF1	Rabbit Monoclonal	Ventana (USA)
P63	Mouse Monoclonal	Ventana (USA)
CD45	Mouse Monoclonal	Ventana (USA)
HMB45	Mouse Monoclonal	Ventana (USA)

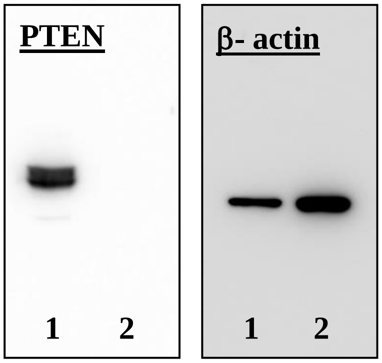

1. MCF10A cells
2. *PTEN*-deficient MCF10A cells

Supplementary Figure 1. PTEN-deficient MCF10A cells are depleted of PTEN protein. MCF10A cells deficient for *PTEN* were generated by a biallelic deletion of the *PTEN* gene. MCF-10A maternal cells (lane 1), and PTEN-deficient MCF10A cells (lane 2) were harvested and processed for western blotting. PTEN expression is completely absent in the *PTEN*-deficient cells. β-actin was used as a loading control. Blots were cropped from different images as shown in Supplementary Figure 3.

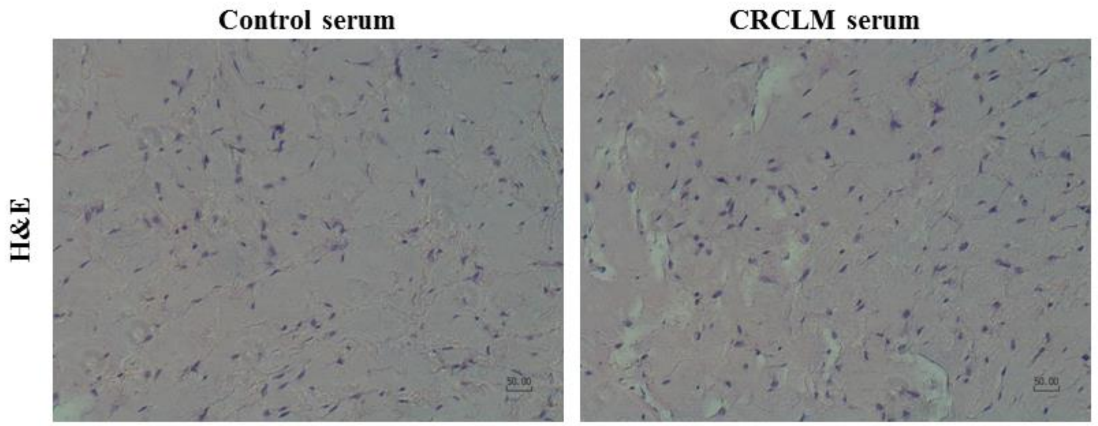

Supplementary Figure 2. Non-mutated MCF10A cells did not transform when exposed to cancer patient sera. MCF10A cells were treated with healthy control or cancer patient sera for 3 weeks. Treated cells were injected subcutaneously into NOD/SCID mice that were followed for 4 weeks for tumors growth. Xenotransplants were excised, fixed in formalin, embedded in paraffin and processed for H&E staining. Representative pictures are shown. Scale bars: 50 µm.

β-actin

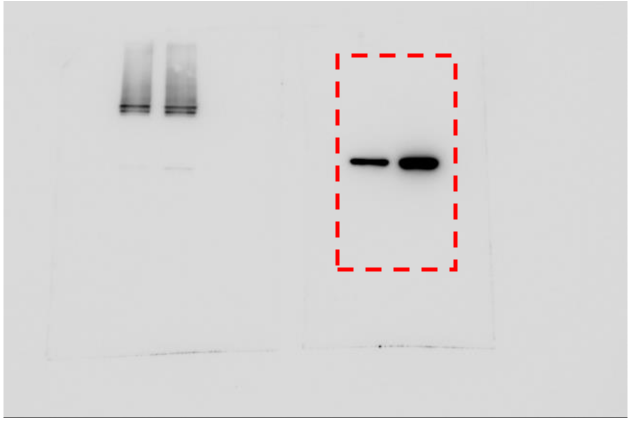

PTEN

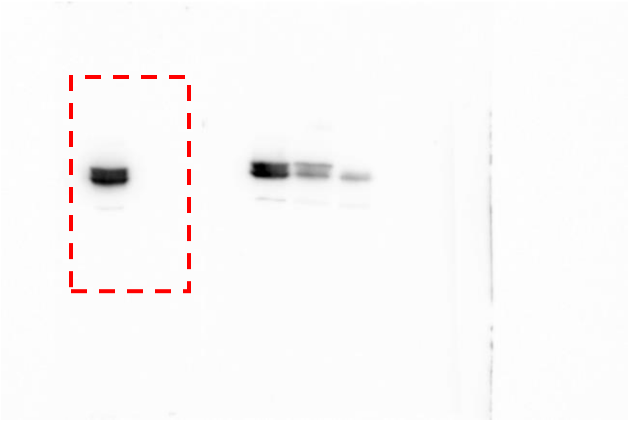

Supplementary Figure 3. Original images of the blots used in Supplementary Figure 1. Cropped regions are shown with dashed line rectangles.
